# Mutations upstream of *fabI* in triclosan resistant *Staphylococcus aureus* strains are associated with elevated *fabI* gene expression

**DOI:** 10.1186/s12864-015-1544-y

**Published:** 2015-04-30

**Authors:** Denis Grandgirard, Leonardo Furi, Maria Laura Ciusa, Lucilla Baldassarri, Daniel R Knight, Ian Morrissey, Carlo R Largiadèr, Stephen L Leib, Marco R Oggioni

**Affiliations:** Neuroinfection Laboratory, Institute for Infectious Diseases, University of Bern, Bern, Switzerland; LA.M.M.B., Dip. Biotecnologie Mediche, Università di Siena, Siena, Italy; Istituto Superiore di Sanità, Roma, Italy; Quotient Bioresearch, Fordham, UK; Institute of Clinical Chemistry, Inselspital, Bern University Hospital, and University of Bern, Bern, Switzerland; Biology Division, Spiez Laboratory, Federal Office for Civil Protection FOCP, Spiez, Switzerland; Department of Genetics, University of Leicester, Leicester, UK; Current address: The University of Western Australia, Nedlands, WA Australia; Current address: IHMA Europe Sa’rl, Epalinges, Switzerland

**Keywords:** Biocide, Resistance, Triclosan, *fabI*, Microarray, Cross-resistance, Promoter mutation

## Abstract

**Background:**

The enoyl-acyl carrier protein (ACP) reductase enzyme (FabI) is the target for a series of antimicrobial agents including novel compounds in clinical trial and the biocide triclosan. Mutations in *fabI* and heterodiploidy for *fabI* have been shown to confer resistance in *S. aureus* strains in a previous study. Here we further determined the *fabI* upstream sequence of a selection of these strains and the gene expression levels in strains with promoter region mutations.

**Results:**

Mutations in the *fabI* promoter were found in 18% of triclosan resistant clinical isolates, regardless the previously identified molecular mechanism conferring resistance. Although not significant, a higher rate of promoter mutations were found in strains without previously described mechanisms of resistance. Some of the mutations identified in the clinical isolates were also detected in a series of laboratory mutants. Microarray analysis of selected laboratory mutants with *fabI* promoter region mutations, grown in the absence of triclosan, revealed increased *fabI* expression in three out of four tested strains. In two of these strains, only few genes other than *fabI* were upregulated. Consistently with these data, whole genome sequencing of *in vitro* selected mutants identified only few mutations except the upstream and coding regions of *fabI*, with the promoter mutation as the most probable cause of *fabI* overexpression. Importantly the gene expression profiling of clinical isolates containing similar mutations in the *fabI* promoter also showed, when compared to unrelated non-mutated isolates, a significant up-regulation of *fabI*.

**Conclusions:**

In conclusion, we have demonstrated the presence of C34T, T109G, and A101C mutations in the *fabI* promoter region of strains with *fabI* up-regulation, both in clinical isolates and/or laboratory mutants. These data provide further observations linking mutations upstream *fabI* with up-regulated expression of the *fabI* gene.

**Electronic supplementary material:**

The online version of this article (doi:10.1186/s12864-015-1544-y) contains supplementary material, which is available to authorized users.

## Background

Post-genomic research in the past years narrowed down significantly the number of pathway proposed to be suitable as targets for antimicrobial treatment. Based on this, the type II fatty acid biosynthesis pathway shows much promise [1]. One frequently targeted enzyme in this pathway is the enoyl-acyl carrier protein (ACP) reductase enzyme (FabI) as witnessed by the multiple *Staphylococcus aureus* FabI inhibitors in clinical trial [2-5]. This is further underlined by the fact that FabI is the target of the first line anti-tuberculosis drug isoniazid, diazaborines and the biocide triclosan [1,6]. The latter is a synthetic, non-ionic, chlorinated *bis*-phenol and is present in many health care products for both hospital and consumer use [7]. It possesses broad spectrum antimicrobial activity against many Gram-positive and Gram-negative bacteria, some fungi [8], and protozoa including *Plasmodium falciparum* and *Toxoplasma gondii* [8]. Triclosan, unlike other biocides, at low concentrations has a single intracellular target by binding to the active site of the FabI. It forms a stable ternary complex with NAD^+^. Triclosan inhibits FabI by allosterically blocking the active site, and therefore prevents bacteria from synthesising fatty acids, which are necessary for building cell membranes and for division [9].

Several studies have demonstrated that bacteria have both natural and acquired mechanisms of resistance to triclosan. Natural resistance is present to varying degrees in bacterial species, which harbour alternatives to *fabI,* (*fabK*, *fabL* or *fabV*) [10-13]. In addition, biodegradation has been found to occur in different environmental species [14]. The primary mechanism of acquired resistance is due to mutations within the coding region of *fabI*, which decrease affinity of the enzyme to triclosan [9,15-19]. Alternatively, active efflux of triclosan has been described in several Gram-negative species, and is mediated by the resistance-nodulation-division (RND) family of pumps [20]. It has been shown that triclosan can activate the transcriptional regulator SmeT of the SmeDEF efflux transporter in *Stenotrophomonas maltophilia* [21].

In addition to target modification and efflux, also titration of the target enzymes has been found to confer resistance. Recent findings have shown the presence of an additional copy of *fabI*, horizontally transferred from *S. haemolyticus*, in the genome of many *S. aureus* isolates with reduced susceptibility to triclosan [22]. Fatty acid biosynthesis is finely regulated in *S. aureus* by a feed-forward system that globally controls the expression of genes involved in this metabolic pathway and that is dependent on the malonyl-CoA intracellular levels [23,24]. This metabolite was shown to bind to and therefore to inhibit the activity of the transcriptional repressor FapR [23]. FapR is a homodimeric repressor highly conserved in Gram positive organisms that has been characterized for its inhibitory function of the expression of genes involved in the fatty acids and phospholipids biosynthetic pathways [23,24]. Among others, FapR was shown to directly interact with the promoter of *fabI* and to physiologically regulate its expression [24]. In analogy to the isoniazide resistance conferring mutation in *Mycobacterium tuberculosis* [6], increased amount of the FabI enzyme has been described in triclosan resistant *S. aureus* strains [16], however no gene expression data are available to sustain this finding. Furthermore, increased levels of *fabI* expression have been observed in *in vitro* adapted *S. aureus* derivatives, with a possible role of promoter mutations in some of these strains [25]. Therefore, the aim of this study was to further characterize mechanisms of resistance in a cohort of previously described *S. aureus* clinical isolates and *in vitro* selected mutants with reduced susceptibility to triclosan by sequencing the putative promoter region of *fabI* and by evaluating the levels of gene expression using microarray analysis.

## Results

### Promoter sequence analysis

**Figure 1:**
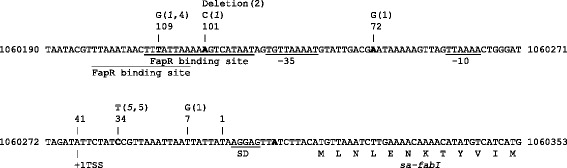
Mapping of mutations in the intergenic region upstream *sa-fabI*. The *sa-fabI* upstream region from the *S. aureus* Mu50 genome (GenBank ID: BA000017) is reported. Nucleotides in which mutations have been identified are marked in bold. The positions of the mutations are reported with respect to the first nucleotide preceding the start codon of *sa-fabI* and numbered backwards. The nucleotide substitution is described above the mutation position together with the number of clinical isolates (italicised number) and mutant strains carrying that particular mutation. ATCC6538 *sa-fabI* upstream region sequence is identical to Mu50, while the naturally occurring polymorphisms identified in the *wt* strains RN4220 (A92T; GenBank ID: AFGU01000045), ATCC25923 (A213T, A188C; GenBank ID: CP009361), and MW2 (T224A; GenBank ID: BA000033) with respect to the Mu50 sequence are not reported as they do not affect triclosan susceptibility. The putative −35 and −10 consensus sequences, identified by BPROM, are underlined. The consensus of the transcriptional repressor FapR recognition sequence is reported as mapped in RegPrecise (underlined) [26] or as previously reported by alignment with the experimentally determined one in the *fapR* upstream region (dotted underlined) [23,24]. The transcriptional start site (+1TSS) as identified by RNAseq [50] and the ribosomal binding site (SD) are also reported.

Seven out of thirty-eight (18%) triclosan resistant *S. aureus* clinical isolates sequenced were found to have polymorphisms in the *fabI* upstream region (Additional file 1: Supplementary data S1). Sequence data showed that the C34T substitution was the most frequent SNP (5 strains), while the SNPs A101C and T109G were found only once (Table 1, Figure 1). The latter SNP was found in clinical isolate QBR-102278-1052 where the insertion of an IS256 element upstream the *fabI* gene created an eight bp duplication (AAAAAGTC), which generated the T109G polymorphism (Table 1; Figure 1). No mutations were found in the nineteen triclosan susceptible isolates (Additional file 1: Supplementary data S1). *FabI* promoter mutations were found in 3 out of 9 strains of the group of isolates without any known triclosan resistance marker, 2/15 in the strains with *sa-fabI* mutations and 2/14 in the strains carrying the *sh-fabI* gene. When we previously analysed the *sa*-*fabI* locus in the twenty-three *in vitro* selected triclosan mutant strains, we found that all (23/23) had a mutated *sa-fabI* gene [22]. Now we found that about half (13/23) of these had an additional *sa-fabI* promoter mutation (Table 1; Figure 1; Additional file 1: Supplementary data S1). The majority of the mutations in the *sa-fabI* promoter reflected those found in clinical isolates, except for the A7G and A72G SNPs and the A101 deletion (Table 1; Figure 1). Similar to the clinical isolates, the C34T mutation was the most represented polymorphism (Table 1; Figure 1). In order to identify other genetic determinants possibly involved in triclosan reduced susceptibility, seven mutants were further analysed by whole genome sequencing. These data revealed that only mutations in the *fabI* locus were shared by all strains. MO052 was the only strain analysed by microarray containing additional SNPs, but no obvious associations between these and changes in the transcriptome could be made (Table 2 and Additional file 2: supplementary data S2).

**Table 1:** **Genotypes and phenotypes of** ***in vitro*** **mutants and clinical isolates with** ***fabI*** **promoter mutations**

**Strain**	**Background**	***sa-fabI*** promoter**	***sa*** **-** ***fabI*** ^**‡**^	***sh*** **-** ***fabI*** ^**‡**^	MIC* ^**‡**^	MBC* ^**‡**^	**Comment**
**RN4220**	-	*wt*	*wt*	-	1	2	
MW2	-	*wt*	*wt*	-	0.12	0.12	
**ATCC6538**	-	*wt*	*wt*	-	0.12	0.25	
**MO036**	RN4220	A7G	mutated	-	4	8	
**MO035**	RN4220	C34T	mutated	-	8	8	
MO047	RN4220	C34T	mutated	-	4	8	
MO049	RN4220	C34T	mutated	-	4	8	
MO076	MW2	C34T	mutated	-	4	8	
CR002	ATCC6538	C34T	mutated	-	4	8	
**MO034**	RN4220	A72G	mutated	-	8	8	
MO077	MW2	A101-	mutated	-	4	32	1 bp deletion
d7	ATCC6538	A101-	mutated	-	2	8	1 bp deletion
MO051	ATCC6538	T109G	mutated	-	4	8	
**MO052**	ATCC6538	T109G	mutated	-	8	16	
MO053	ATCC6538	T109G	mutated	-	4	8	
MO055	ATCC6538	T109G	mutated	-	4	8	
QBR-102278-1097	-	C34T	mutated	-	0.25	32	
**QBR-102278-1889**	-	C34T	wt	-	0.5	16	
**QBR-102278-1969**	-	C34T	wt	-	0.25	32	
QBR-102278-2095	-	C34T	wt	-	0.25	32	
QBR-102278-2546	-	C34T	mutated	+	1	64	
**QBR-102278-2363**	-	A101C	wt	+	16	32	
**QBR-102278-1052**	-	T109G	wt	+	0.5	64	IS256 insertion

*MIC and MBC to triclosan are expressed as mg/L. **Polymorphic sites are indicated counting backwards from the *sa-fabI* start site of *S. aureus* Mu50 (GenBank ID: BA000017; position 1060308). Strains analysed by microarray are indicated in bold. ^‡^With the exception of strain QBR-102278-2095, these data have been previously reported [22].

**Table 2:** **Polymorphic changes in the genome of** ***in vitro*** **mutant strains**

Ref. genome positions*		**Annotation**	**RN4220 derived**			**ATCC6538 derived**			**Mu50 derived**
**NCTC8325**	**Mu50**		**MO034**	**MO035**	**MO036**	d2**	**d7**	**MO052**	MO079**
120767		Capsular polysaccharide synthesis enzyme Cap5B					A 592 T – Met 198 Leu		
208900		Pyruvate formate-lyase 1-activating enzyme						G 721 T - Val 241 Phe	
226813		Flavohemoprotein					-TA 953 deletion leading to transcription premature truncation		
784247		Protein traslocase subunit SecG					T 22 A – Leu 8 Ile		
919922		*fabI* upstream region**						T 109 G	
919929		*fabI* upstream region					A 101 deletion		
919959		*fabI* upstream region	A 72 G						
919997		*fabI* upstream region		C 34 T					
920024		*fabI* upstream region			A 7 G				
920098		*fabI* coding region		G 68 C – Gly 23 Ala					
920331		*fabI* coding region	G 301 T – Asp 101 Tyr		G 301 T – Asp 101 Tyr				
920469		*fabI* coding region					T 439 C – Tyr 147 His		
920641		*fabI* coding region				T 611 G – Phe 204 Cys		T 611 G – Phe 204 Cys	
	1060928	*fabI* coding region							T 611 A – Phe 204 Tyr
1139511		serine/threonine-protein kinase PrkC					G 1309 A – Val 437 Ile		
1231020		DNA mismatch repair protein MutL						T 1709 A – Ile 569 Asn	
	1238213	MurD UDP-N-acetylmuramoyl-L-alanyl-D-glutamate synthetase							T 338 G – Ile 113 Ser
	1238872	MurD UDP-N-acetylmuramoyl-L-alanyl-D-glutamate synthetase							C 997 G – Leu 333 Val
1588812		tRNA methylthiotransferase YqeV					G 824 A – Ile 275 Thr		
2335216		HTH-type transcriptional regulator SarV					C 140 A – Val 47 Gly		
2774594		Ica operon transcriptional regulator, IcaR					-TCTTTTGTCA 417 deletion leading to transcription premature truncation		

*The nucleotide position refers to the genome of *S. aureus* NCTC8325 (accession NC_007795) or Mu50 (accession NC_002758). Polymorphic sites of *fabI* promoter region are indicated counting backwards from the *sa-fabI* start site of *S. aureus* Mu50 (GenBank ID: BA000017; position 1060308). **Despite not having *fabI* promoter mutations the d2 and MO079 mutants have been included in the analysis to check for the presence of mutations (not only in the *fabI* coding sequence) playing a possible role in triclosan reduced susceptibility.

### Gene expression analysis

#### Laboratory mutants

Transcriptomic differences between triclosan susceptible *wild type* strains and their resistant isogenic mutants were analysed by microarrays. Four pairs with different mutations in the promoter regions were compared (Table 3 and Table 4). Strains with the following promoter mutations were analysed: A7G in MO036 and A72G in MO034 were mutations documented only in laboratory strains, but not in clinical isolates. C34T in MO035 was the most frequently observed mutation in both laboratory strains and clinical isolates. Finally, we also analysed T109G, the second most frequent mutation in the laboratory strains, which was also identified in a clinical isolate (Table 3, Figure 1). *fabI* was up-regulated in three of the four tested laboratory strains, with a change in fold expression between 3.47 and 4.97 (Table 3 and Table 4). The *fabI* gene was the highest up-regulated in both MO035 and MO052 (Additional file 2: Supplementary data S2). MO034 was characterized by a higher number of up- and down-regulated genes (Additional file 2: Supplementary data S2) with *fabI* being the third highest regulated probe. In contrast the strain MO036, carrying the A7G mutation, was not found to have an up-regulated *fabI* expression. In this strain, only two genes, not previously described to be involved in the development of triclosan resistance, were slightly up-regulated (Additional file 2: Supplementary data S2). No further significant association were observed between polymorphisms retrieved with the whole genome sequencing and up- and down-regulation of corresponding genes in mutant strains.

**Table 3:** **Overview of the changes in gene expression in laboratory mutants and clinical isolates**

**Comparison strains**	**Promoter mutation**	**Up-regulated genes**	**Down-regulated genes**	**Fold increase** ***fabI*** **expression**
MO034 vs RN4220	A72G	154	125	4.97
MO035 vs RN4220	C34T	7	-	3.92
MO036 vs RN4220	A7G	2	-	-
MO052 vs ATCC6538	T109G	3	20	3.47
QBR-102278-1889 vs ATCC25923	C34T	76	18	4.86
QBR-102278-1969 vs ATCC25923	C34T	78	36	6.44
QBR-102278-2363 vs ATCC25923	A101C	122	28	4.26
QBR-102278-1052 vs ATCC25923	T109G	198	8	47.6

**Table 4:** ***fabI*** **/** ***gyrA*** **ratio of laboratory and clinical strains**

**Strains**	**Log**_**2**_ ***fabI/gyrA*** ratio*	***t*** **test vs** **parental strain**	***t*** **test vs ATCC 6538**	***t*** **test vs ATCC25923**
Laboratory strains				
RN4220 (n = 6)	1.69			
MO034 (n = 5)	2.49	0.04		
MO035 (n = 5)	3.37	0.0002		
MO036 (n = 3)	1.41	ns		
MU50 (n = 5)	−0.28			
ATCC6538 (n = 4)	0.14			
MO052 (n = 4)	2.14	0.0002		
ATCC25923 (n = 4)	−0.51			
Clinical strains				
QBR-102278-1052 (n = 4)	4.22		<0.0001	<0.0001
QBR-102278-1889 (n = 4)	1.25		0.0026	0.0137
QBR-102278-1969 (n = 4)	1.56		0.0002	0.0059
QBR-102278-2363 (n = 4)	1.16		0.004	0.016
QBR-102278-1016 (n = 3)	0.23		ns	ns
QBR-102278-1027 (n = 4)	0.13		ns	ns
QBR-102278-2628 (n = 5)	0.03		ns	ns

*Log_2_ ratio was independently determined for each chip using the data before stage-wise quantile normalization, by subtracting the Log_2_ mean *gyrA* values obtained from the eight probes for *gyrA* printed on the chip from the Log_2_ mean *fabI* values from the two probes. Ratio was then further averaged for the number of chips used by strains (n). Student’s unpaired *t*-test was performed by comparing the mutants to their parental strains in the case of laboratory strains, or to two reference strains in the case of clinical isolates. ns = not significant.

#### Clinical isolates

A comparison between triclosan resistant clinical isolates containing mutations in the *fabI* promoter and sensitive prototypical strains was also performed. Four clinical isolates with reduced triclosan susceptibility (Table 3) were compared to the prototypical, triclosan susceptible *S. aureus* strain ATCC25923 (MIC and MBC of 0.06 and 1 mg/L respectively). Two of these strains (QBR-102278-1889 and QBR-102278-1969) had the same *fabI* promoter mutation (C34T) as the laboratory strain MO035. One strain (QBR-102278-1052) had the mutation T109G, also found in MO052. Finally, one strain (QBR-102278-2363) showed a mutation (A101C) in a region deleted in two laboratory strains, which were however not tested by microarrays (Table 3, Figure 1). A higher number of up- or down-regulated genes were found in these clinical isolates than in laboratory strains, except MO034. This was expected, since the comparison could not be made between isogenic strains. Furthermore, the genetic diversity of these strains may be augmented by the presence of plasmids not present in ATCC25923. Nevertheless, in all clinical strains, *fabI* was up-regulated, from 4.26 to 47.6 fold (Table 3, Table 4, and Additional file 2: Supplementary data S2). Also the Log_2_*fabI*/*gyrA* ratios were found to be significantly higher in the four clinical strains with decreased sensitivity to triclosan when compared to the two reference strains ATCC6538 and ATCC25923 (Table 4). This was in contrast to 3 triclosan susceptible strains (QBR-102278-1016, QBR-102278-1027, and QBR-102278-2628; described in Additional file 1: Supplementary data S1), which showed ratios comparable to the reference strains (Table 4). Quantitative real-time PCR confirmed a statistically significant increase of comparable entity in the expression of the *fabI* gene of clinical isolates when compared to reference strains (using *gyrA* as housekeeping control). The increase was for the *fabI* gene of QBR-102278-1052, QBR-102278-1889, QBR-102278-1969, and QBR-102278-2363 respectively 36.9, 9.0, 15.6, and 7.7 fold compared to ATCC6538 and of 9.9, 2.4, 4.2, and 2.1 fold when compared to ATCC25923. QBR-102278-1052, the strain with the highest up-regulation of *fabI* and the highest MBC was also characterized by the presence of an IS256 element upstream of the promoter. Other possible mechanisms of triclosan resistance in these clinical strains were evaluated by identifying the genes commonly up- or down- regulated in the four clinical isolates. 37 genes were found to be up-regulated in all 4 strains (Tables 5, 6 and Additional file 3: Supplementary data S3). Furthermore, 6 genes were down-regulated. Apart from *fabI*, all genes found to be up- or down- regulated were of chromosomal origin, meaning that, at least in the four clinical isolates tested in the present study, triclosan resistance is not plasmid-mediated at the level of gene expression. Gene ontology (GO) annotations were available for 29 genes of all 43 differently regulated genes (Additional file 3: Supplementary data S3). Genes involved in transport (8 genes) or in membrane structure/metabolism (12 genes) were the most represented. These intrinsic differences in bacterial membrane properties could influence triclosan tolerance independently from *fabI*, for example by altering triclosan trafficking through the membrane. However, we did not find genes coding for efflux pumps or known antibiotic/biocide resistance genes to be up-regulated (Tables 5 and 6).

**Table 5:** **Genes up-regulated in triclosan resistant** ***S. aureus*** **clinical isolates with respect to ATCC25923**

	**QBR-102778**			
**Genes**	**-1889**	**-1969**	**-2363**	**-1052**
cycA / putative D-serine/D-alanine/glycine transporter	2.5	3.2	4.7	6.5
dltD / putative lipoteichoic acid biosynthesis protein	2.9	2.3	2.3	8.9
**fabI / enoyl-acyl-carrier-protein reductase (NADH)**	**4.8**	**6.4**	**4.3**	**47.6**
fruA / PTS transport system, fructose-specific IIABCcomponent	5.3	4.9	11.1	11.1
gyrA / DNA gyrase subunit A	2.6	2.4	2.2	3.4
hlb / phage protein	3.2	2.6	4.9	10.5
isaB / hypothetical protein	2.8	2.4	2.5	5.4
mprF / putative membrane protein	4.5	2.2	6.8	8.3
murA1 / putative UDP-N-acetylglucosamine1-carboxyvinyltransferase	4.6	4.3	8.3	17.1
rho / transcription termination factor	2.2	2.2	3.3	7.4
rir1 / ribonucleoside-diphosphate reductase alphachain	2.4	2.1	2.4	4.0
SATW20_19350 / phage protein	6.0	5.9	10.6	17.9
SAV0240 / SATW20_02420 / flavohemoprotein	10.6	18.4	26.0	13.7
SAV0348 / SATW20_04160 / hypothetical protein	2.1	2.1	3.5	2.3
SAV0465 / SATW20_05330 / putative exported protein	7.0	3.8	7.7	13.7
SAV0663 / SATW20_07380 / conserved hypothetical protein	4.8	5.4	9.0	14.1
SAV0699 / SATW20_07740 / putative phosphofructokinase	2.5	2.6	4.7	3.9
SAV0944 / SATW20_09440 / thioesterase superfamily protein	3.6	2.6	8.9	2.0
SAV1356 / SATW20_13570 / sodium:alanine symporter family protein	2.1	2.3	2.9	4.3
SAV1573 / SATW20_15690 / putative exported protein	3.0	2.2	4.6	5.5
SAV1853 / SATW20_18470 / putative membrane protein	2.7	2.5	3.0	2.9
SAV1914 / SATW20_19090 / putative oxygenase	3.9	2.7	6.6	6.6
SAV1947 / hypothetical protein	22.4	6.3	28.3	9.3
SAV2032 / SATW20_20150 / membrane anchored protein	3.3	2.2	3.3	3.7
SAV2184 / SATW20_23220 / putative membrane protein	4.8	4.1	7.2	6.0
SAV2253 / SATW20_23870 / xanthine/uracil permease family protein	6.2	3.4	3.8	3.7
SAV2253 / SATW20_23870 / xanthine/uracil permeases family protein	4.3	2.2	2.4	3.0
SAV2335 / SATW20_24670 / putative membrane protein	2.5	2.3	3.8	2.6
SAV2368 / SATW20_25000 / hypothetical protein	5.1	3.2	6.6	6.7
SAV2368 / SATW20_25000 / putative lipoprotein	9.8	6.6	16.1	10.0
SAV2383 / SATW20_25130 / putative exported protein	3.0	3.3	4.0	7.4
SAV2403 / SATW20_25330 / putative nitrite transporter	3.5	2.4	2.8	6.1
SAV2404 / SATW20_25340 / putative membrane protein	6.7	4.9	6.2	3.3
SAV2412 / SATW20_25420 / ABC transporter ATP-binding protein	10.1	8.5	16.3	25.5
SAV2413 / SATW20_25430 / ABC transporter permease	6.2	4.9	9.6	18.4
SAV2413 / SATW20_25430 / transport system membrane protein	3.9	3.4	4.7	8.0
SAV2414 / SATW20_25440 / extracellular solute-binding lipoprotein	2.4	2.2	3.1	5.0
scn / staphylococcal complement inhibitor SCIN	64.7	63.5	47.7	85.1
trap / signal transduction protein TRAP	10.6	9.5	16.7	21.7
xpt / putative xanthine phosphoribosyltransferase	7.8	6.5	12.5	12.9

**Table 6:** **Genes down-regulated in triclosan resistant** ***S. aureus*** **clinical isolates with respect to ATCC25923**

	**QBR-102778**			
**Genes**	**-1889**	**-1969**	**-2363**	**-1052**
fda / fructose-bisphosphate aldolase class I	0.03	0.02	0.03	0.04
SATW20_01020 / putative hydratase	0.17	0.15	0.29	0.30
SATW20_28340 / putative N-acetyltransferase	0.11	0.07	0.06	0.03
SAV0801 / hypothetical protein	0.17	0.16	0.15	0.13
SAV2515 / SATW20_26360 / transmembrane protein smpB	0.38	0.46	0.24	0.38
SAV2643 / SATW20_27810 / putative membrane protein	0.45	0.38	0.35	0.31

## Discussion

Several mechanisms inducing reduced triclosan susceptibility have been described in both Gram-positive and Gram-negative organisms [9-22]. Amongst them, mutations in the coding regions of the *fabI* gene or an increase in its expression level have been related to triclosan resistance [9,15-19].

The analysis of the *fabI* upstream region in triclosan resistant clinical isolates and *in vitro* selected mutant strains revealed the presence of nucleotide changes with respect to triclosan susceptible strains. Interestingly three mutations (T109G, A101C, and A101-del) were found to occur within the FapR DNA recognition sequence. These mutations are likely to interfere with FapR binding, therefore reducing its inhibitory effect on *fabI* transcription. More importantly, the thymine in the 109 position was found to be highly conserved among the FapR DNA recognition sequences [26], while the 101 adenine was previously showed to be specifically recognized and bound by the Arg56B residue of FapR [23]. It is noteworthy that the A72G mutation, present only in a laboratory mutant strain, was found to occur between the predicted −35 and −10 promoter sequences. On the other hand no clear indication on the mode of action of the C34T mutation, located 7 bp downstream the transcription start site, could be found. The high frequency of this mutation in clinical isolates points to an important regulatory effect, which might be hypothesised to be linked to post-transcriptional regulation. A hypothesis strengthened by the high level of sequence identity, including a complete match of the sequence preceding C34, between the staphylococcal *fabI* 5-prime UTR to *Enterococcus faecalis* [27]. However further studies will be required to determine the exact nature of such regulatory events.

Our data indicate that mutations in the *fabI* upstream region were not always found to be associated to other previously described triclosan resistance mechanisms. Indeed, *fabI *promoter mutated strains were found either alone or associated to the presence of the *sh-fabI* gene or mutations in the *sa-fabI*. In particular we found three triclosan resistant clinical strains with mutations in the *fabI* promoter, but not in the coding region. In two of these strains analysed by microarray an up-regulation of the *fabI* gene was also observed suggesting that these promoter mutations in *fabI* may induce overexpression and participate in triclosan resistance. Although having mutation in the promoter region, the strain MO036 didn’t display higher level of *fabI* gene expression. The most plausible cause of triclosan resistance in this strain is the observed mutation in the coding region of the gene. However, *isaA*, overexpressed in MO036, could also be an additional strategy contributing to triclosan resistance, since its involvement has been recently mentioned for fusidic acid resistance, by altering cell wall metabolism and therefore cell properties [28].

Previous experiments showed that mutations in the coding region of *fabI* varied between mutant strains and clinical isolates [22]. In contrast, there was good overlap between *in vitro* selected *fabI* promoter mutations and those of clinical strains in the present study (Figure 1). On the other hand, phenotypic differences were evident between mutants and clinical isolates carrying *fabI* promoter mutations (Table 1). Indeed mutant strains showed higher MICs values and lower MBCs values with respect to clinical isolates. The higher MICs could be explained by the technical constrain imposed by the mutant selection method [22] in which the active multiplying cells need to be grown in presence of triclosan concentrations sufficient to isolate resistant mutants from *wt* strains. On the other hand *in vivo* concentrations of triclosan could be lower, potentially transient and acting on bacteria with greater generation times. These three aspects, not reproducible in *in vitro* conditions, could have allowed for a different selection of triclosan resistant mutants in the natural environment, including in the human host. Such phenotypic differences were also reflected by the absence of *in vitro* generated mutants showing the sole *fabI* upstream mutation (Table 1) confirming that our laboratory mutant selection strategy couldn’t select triclosan resistant strains without mutations in the *fabI* coding region, while clinical isolates could evade triclosan bactericidal activity by increasing the baseline expression of *fabI* through the sole promoter mutations. However these divergences do not indicate any reduction of fitness neither in the lab strains nor the clinical isolates [29].

The technical constrain for selecting high MIC mutants in vitro is due to the drug concentration used in the screening of active multiplying cells. Probably in vivo concentrations drug concentrations can be lower and potentially transient and the bacterial generation time is greater. These three aspects could allow a different selection of mutants in vivo. Considering lower and transient drug concentration and possibly greater generation time, it is possible that an increased baseline expression may allow for out-titration of killing effect.

Microarray analysis confirmed that in all clinical strains and the majority of lab mutants, promoter mutations were associated with an up-regulation of *fabI* transcription (Tables 4, 5 and 6). Increased expression of *fabI* gene has been described in triclosan-resistant *S. aureus* clinical isolates [16], *S. epidermidis* mutants [30], laboratory mutants of *S. aureus* [25], *E. coli* and *Salmonella* [31-33]. Promoter mutation has never been linked to *fabI* over-expression despite mutation in the *fabI* upstream region were previously identified in adapted *S. aureus* USA300 isolates [25]. Interestingly, the exposure of *S. aureus* [34], *Salmonella enterica* or *E. coli* [35] to triclosan does not necessarily lead to *fabI* up-regulation. One of our clinical strains (strain QBR-102278-1052), distinguished itself by a very high level (approx. 50 fold up-regulation) of *fabI* expression. It is of notice that quantitative real-time PCR confirmed the particularly high *fabI* expression of QBR-102278-1052 isolate. In this clinical strain, the insertion of an additional *sh-fabI* allele was also documented. Still, the observed increase in signal on the microarray was exclusively due to *sa-fabI* hybridization, since the two oligonucleotide probes printed on the array were able to discriminate *sa-fabI* from *sh-fabI*. Rather, we found an IS256 insertion sequence element 114 bp upstream of and in the same direction as the *fabI* gene in this strain. Multiple copies of IS256, not associated with any resistance genes, have been found in the chromosome of *S. aureus* [36], but also in many strains of *Enterococcus faecalis* and *E. faecium* [37]. The formation of a potent hybrid promoter containing IS256 could be a new additional mechanism leading to high level of *fabI* expression and decreased susceptibility to triclosan, which however remains to be proven experimentally. This hypothesis would be supported by the observation of a high level resistance to methicillin and fluoroquinolones in *S. aureus* induced by the insertion of IS256 upstream of the *llm* and *norA* genes respectively [38,39]. A similar mechanism was also described in *Staphylococcus sciuri*, in which methicillin resistance was linked to the insertion of IS256 upstream of the gene coding for a *mecA* homolog [40].

Our microarray analysis of triclosan-resistant clinical isolates did not reveal any efflux-mediated resistance mechanism, neither chromosomally-encoded, nor plasmid-mediated. This is in agreement with previous studies showing that there was no significant increase in the triclosan MBC for *S. aureus* strains carrying plasmid-borne *qac* genes coding for multidrug efflux pumps [41]. Similarly, over-expression of the chromosomal *norA* multidrug transporter gene did not lead to triclosan resistance [42]. Efflux-mediated resistance to triclosan is however not to be excluded in Gram- positive bacteria. Indeed, it has been recently shown that, out of 21 *S. haemolyticus* clinical strains, an inhibition of efflux pump activity by carbonyl cyanide-m-chlorophenylhydrazone (CCCP) significantly decreased triclosan MIC in four strains [43] suggesting the possible presence of a still unidentified efflux system with triclosan as a substrate. Despite this fact, efflux-mediated triclosan resistance to date still remains restricted to Gram-negative species [20,21]. Other mechanisms inducing an alteration of membrane metabolism, structure or trafficking in *S. aureus* could not be excluded. This hypothesis would be supported by the identification of several upregulated genes involved in such mechanisms in clinical strains or *isaA* up-regulation in the laboratory mutant MO036.

Apart from the *fabI* gene or efflux pumps, evidence for other mechanisms of resistance to triclosan is scarce. Prolonged exposure of MRSA to triclosan-impregnated silicon elastomer resulted in the selection of small colony variants resistant to triclosan, but the underlying mechanism is still not understood and may be non-specific, involving a reduction in energy generation and/or transport and the down-regulation of functions such as cell wall synthesis [44]. High-throughput methods (proteomics, genomics) have been recently applied to laboratory strains of *S. typhimurium* and *E. coli* grown in absence of triclosan in order to reveal inherent mechanisms of resistance to triclosan based on changes in genes or proteins expression [32,33,45]. Apart from the consistent up-regulation of *fabI*, relatively few new mechanisms were proposed. It has been postulated that the increased expression of dehydrogenases and oxidoreductases using NAD^+^ as a cofactor, could bind and capture triclosan, reducing its effective intracellular concentration [32,45]. In the present study, apart from *fabI*, only one putative flavohemoprotein (SATW20_02420) containing identified binding sites for NAD or FAD was highly up-regulated in the clinical strains. In most cases no complete operon was found to be over-expressed in the triclosan resistant clinical isolates. Exceptions are the co-transcribed *fruA* and *fruB* genes and the three genes for an amino acid ABC uptake system (SAV2412-4). This is in accordance with previous data, which had shown that exposure of *S. aureus* to triclosan leads to the de-regulation of branched amino acid uptake and carbohydrate metabolism including changes in expression of *fruA, fruB* and *xprT* [34]. Albeit the partial overlap of data between our characterisation of resistant isolates and the work on triclosan toxicity, no clear metabolic correlation can be drawn, which links triclosan and fatty acid metabolism to the observed changes in gene expression. In that respect, the current analyses of the six clinical isolates devoid of any resistance markers could also provide further insights, especially if *fabI* turns out not to be overexpressed in these strains.

To our knowledge, this is the first extensive microarray analysis comparing both *in vitro* generated mutants and clinical isolates of *S. aureus* resistant to triclosan. The comparison of the mutants and their parental strains enables us to link genetic variations to phenotypic changes more directly. For clinical strains, comparisons between resistant strains and a prototypical strain do not allow such direct conclusion and also relate to the choice of the prototypical strain. These results were therefore confirmed by determining *fabI*/*gyrA* ratio. To definitely challenge the hypothesis that mutations in the promoter of *fabI* lead to overexpression of the gene and consequently to a reduced susceptibility, prospective genetic manipulation would have been a more direct approach. Nevertheless, whole genome sequencing revealed in three out of four laboratory mutant tested by microarray, that the only mutation susceptible to explain change in *fabI* expression was located in *fabI* promoter region. Furthermore, the fact that the same *fabI* promoter mutations were found in all clinical strains with overexpression of their *fabI* gene, indirectly provide the evidence for the involvement of such mutations in *fabI* up-regulation and possibly in triclosan reduced susceptibility.

## Conclusion

In conclusion, molecular changes in the promoter region of *fabI* were identified together with *fabI* over-expression and triclosan resistance. As such this overexpression of *fabI* has the potential to determine cross-resistance to novel compounds in clinical trial [2-5]. This adds to the recently described mutations within *fabI* selected by the novel compound AFN-1252 which confer cross-resistance to triclosan [46]. It should be noted that in *S. aureus fabI* up-regulation acts almost always in addition to the other triggers of triclosan resistance, such as mutations in coding regions of the *fabI* gene or the insertion of an addition allele derived from *S. haemolyticus*. Importantly, we could not link triclosan resistance in staphylococci to the presence or over-expression of efflux systems, either of plasmid or chromosomal origin, a mechanism known to contribute to resistance in Gram-negative organisms [47] or to the resistance to any other antimicrobial drug [48]. In addition we have solid evidence of the *fabI* up-regulation in four triclosan resistant clinical isolates for which the intrinsic genetic diversity between clinical isolates so far had eluded comparison of gene expression. Indirect evidences support the relationship between *fabI* overexpression and the presence of mutations mapped in the FapR binding domain or other regions of the predicted *fabI* promoter region. This hypothesis is further strengthened by the finding of the same mutations in *fabI-*overexpressing laboratory mutants, which, most importantly, were shown to not carry any other mutation in their genome that could be associated to triclosan resistance. This combined data in laboratory mutants and clinical isolates opens new avenues to explore mechanisms of triclosan resistance in *S. aureus*.

## Methods

### Bacterial strains

Sixty-five *S. aureus* strains with reduced susceptibility to triclosan were previously selected from a collection of 1602 clinical isolates by performing standard MIC and MBC assays [22,48,49]. Of these, fifteen strains with mutations in the *fabI* coding sequence, fourteen with an additional chromosomal *sh-fabI* allele (from *S. haemolyticus*)*,* and nine without any known triclosan “resistance” marker were investigated in this work for *fabI* promoter mutations (Additional file 1: Supplementary data S1). As a control nineteen triclosan susceptible isolates were included in the analyses (Additional file 1: Supplementary data S1). In addition twenty-three independent mutants (Additional file 1: Supplementary data S1), with reduced susceptibility to triclosan, were also analysed. These strains have been previously selected by single-exposure of ATCC6538, MW2, and Mu50 reference strains to 0.5 mg/L of triclosan in solid medium or by multiple step-growth on liquid medium with increasing triclosan concentrations (from 0.25 mg/L to 4 mg/L) as in the case of the RN4220 laboratory strain [22].

No ethical approval was required to obtain the isolates used in the study. No ethical approval was required to use the clinical isolates in this study. Isolates were obtained during routine microbiological investigations and were not part of a clinical trial.

### Molecular analysis

The upstream region of the *fabI* gene was amplified in the fifty-seven clinical isolates and in the twenty-three laboratory mutants. DNA was amplified using standard PCR conditions and the following primers: 5′-ATCATCTTCGTGCGTATTATC-3′ and 5′-TTCAAGCTCTTTACGGCTA-3′ (Eurofins MWG Operon, Germany). PCR products were sequenced by the Sanger method (Eurofins MWG Operon, Ebersberg, Germany). A selection of *S. aureus fabI* upstream sequences has been deposited in GenBank (accession nos. KF583951- KF583970). The putative −35 and −10 sequences have been predicted using the BPROM tool (http://www.softberry.com/berry.phtml), while the FapR recognition sequence was mapped by mean of the data available on the RegPrecise database [26]. The transcriptional start site was identified by direct visualisation of RNA-seq alignment data retrieved from the NCBI Sequence Read Archive (SRA, http://www.ncbi.nlm.nih.gov/Traces/sra/). More precisely the illumina HiSeq data were previously generated by sequencing of the RNA extracted from a *S. aureus* Newman strain at the early log phase (SRA Experiment ID: DRX011556, SRA Sample ID: DRS011392) [50]. Whole-genome sequencing was performed by the Institute of Applied Genomics (University of Udine, Italy) using an Illumina Genome Analyzer II platform (Illumina, San Diego, California, USA) for the *in vitro* selected mutants d2, d7, MO034, MO035, MO036, MO052, MO079 and their isogenic *wild type* strain RN4220, ATCC6538, and Mu50 (Table 2). Mutants to be sequenced have been selected on the basis of their *fabI* promoter sequence in order to analyse one strain for each one of the previously identified mutations. Strains d2 and MO079 have been also included in order to check for genetic changes, other than *sa-fabI* mutations, possibly related to their triclosan reduced susceptibility phenotypes. Sequences of both *wt* strains and mutant strains, were aligned to the reference genome of *S. aureus* NCTC8325 (accession NC_007795), except for Mu50 and MO079 that were aligned to the Mu50 genome (accession NC_002758), using the Mosaik Assembler suite (The MarthLab, Boston College, Massachusetts, USA). Single nucleotide polymorphisms (SNPs), insertions and deletions (INDELs) were retrieved with VarScan software [51]. SNPs and INDELs of the *wt* strains obtained from the alignment with the GenBank reference genome were subtracted from those found by aligning the mutant strain with the reference.

### Statistical analysis

Fisher’s exact test was applied to assess if the differences in the number of clinical isolates with *fabI* promoter mutations among the three groups of strains defined by known triclosan resistance marker were statistically significant.

### Gene expression analysis

#### Array design and production

Probe design was performed by the CustomArray Design Service. Probes of 35–40 bp length were selected based on melting temperature (T_m_), complexity, secondary structure, GC-content, and specificity. A total of 12’000 capture probes were finally used. Furthermore, the array also contained quality control spots, non-specific probes derived from phages, plants, virus and bacteria, as well as empty, oligonucleotide-free spots. The entire genomes of Mu50 and TW20 were covered in the microarrays, plus additional elements as listed in the Additional file 4: Supplementary data S4. Arrays were synthesized on a CustomArray Synthesizer (CombiMatrix, Mukilteo, WA) and quality tested using the standard protocols provided by the manufacturer.

#### Bacterial growth

*S. aureus* strains were grown overnight in 10 ml tryptic soy broth (TSB) at 37°C at 80 rpm. The cultures were diluted 1:100 in pre-warmed TSB and grown to logarithmic phase (OD_570_ = 0.6). 2 ml of each culture (1–5 × 10^8^ colony forming units) was harvested in 4 ml of RNAprotect® reagent (Qiagen), incubated for 5 min at room temperature and centrifuged for 10 min at 5000 × g. The pellet was then processed directly for RNA extraction or stored at −80°C for later processing.

#### RNA purification

Total RNA was extracted using RNeasy Mini Kit (Qiagen), according to the manufacturer’s instructions, using 50 U recombinant lysostaphin (Sigma) followed by incubation for 5 minutes with 1 ml of hot Qiazol (Qiagen) to lyse bacteria. Bacteria were further disrupted by vibration with 50 mg of acid-washed glass beads (Sigma) using a Mickle Vibratory Tissue Disintegrator (Mickle Laboratory Engineering) at maximum speed. Contaminating DNA was removed using DNA-free™ Kit (Applied Biosystems) and RNA quality tested on an Agilent 2100 Bioanalyzer (Agilent Technologies). RNA concentration and purity were determined by Nanodrop® ND-1000 spectrophotometer (Thermo Scientific). For each strain, at least 4 RNA samples were prepared from independent cultures.

#### RNA labelling and fragmentation

Isolated, unamplified RNA was labelled with Cy5, using ULS™ Labeling Kit for CombiMatrix arrays (Kreatech Biotechnology), according to the manufacturer’s instructions. RNA was finally fragmented with RNA Fragmentation Reagents (Ambion®).

#### Array hybridization

12 K Customarrays were hybridized with 2 μg of labelled, fragmented RNA, according to information provided by the manufacturer (Customarray/Combimatrix Incorporated). Microarrays were scanned using the Packard ScanArray4000 array scanner and software (ScanArray, version 3.1, Packard BioChip Technologies). All arrays were scanned with incremental laser power from 15 to 100%. Data were extracted with Microarray Imager software (version 5.8.0, Combi Matrix) and spot intensity expressed as median intensity.

#### Data analysis

Scanning data with similar median fluorescence intensity were chosen for analysis. Fluorescence values of spots with maximal intensity (signal saturation) at the chosen laser intensity were extrapolated by linear regression, using values gathered from the two next lower laser intensities.

#### Gene filtering

Non-specific binding was determined from fluorescence values of all non-specific probes. The cut-off for specific binding was set as the upper 95% confidence interval of the mean signal intensity of the non-specific probes. For each comparison, probes were excluded when the mean values for both strains to be compared were under the determined cut-off. For comparisons involving *in vitro* generated mutants derived from plasmid-free strains RN4220 or ATCC6538, analysis was performed using only probes sets gathered from the genomes of *S. aureus* TW20 and Mu50.

#### Data transformation, normalization and analysis

The fluorescence values were log2 transformed. For each set of comparison, stage-wise quantile normalization was performed, using a script written in the statistical computing environment of R (R Development Core Team, 2011), according to Deshmukh et al. [52]. Significantly differentially regulated genes were determined by using the Significance Analysis of Microarrays method (SAM, Excel Add- in version 4.0) originally developed at Stanford University lab [53]. For each comparison, the delta value was set to obtain a conservative median false discovery rate (FDR) of 1% and the fold change cut-off value was set to 2. For investigating common up- or down- regulated genes in the 4 triclosan resistant clinical strains, the FDR value was set to 5%.

#### Quantitative real-time PCR

Reverse transcription of total RNA to single-stranded cDNA was performed on selected laboratory strains and clinical isolates using the High Capacity RNA-to-cDNA Kit (Applied Biosystems). Quantitative real-time PCR was carried out using SYBR® Green PCR Master Mix (Applied Biosystems) and the reactions were performed in triplicate, according to the manufacturer’s instructions, using a 7500 Fast Real-time PCR System (Applied Biosystems). The *fabI* gene was amplified using the primers 5′-GTCCAATCCGTACATTAAGTGCA-3′ and 5′-TCACCTGTAACGCCACTTGATAA-3′. The results were normalised to the housekeeping gene *gyrA* amplified using the primers 5′-ACGTCAACGTATTGTTGTCACTG-3′ and 5′-TTACGCACATCAATAACGACACG-3′. Transcription levels were determined using the 2^-ΔΔCT^ method [54].

### Availability of supporting data

The data sets supporting the results of this article are available in the ArrayExpress repository, (http://www.ebi.ac.uk/arrayexpress/) under accession numbers A-MEXP-2362 (*S. aureus* array design) and E-MTAB-2127 (microarray raw results).

## Additional files

Additional file 1:
**Supplementary data S1: Relevant information of** ***S. aureus*** **strains analysed.**
Additional file 2:
**Supplementary data S2: Statistically significant genes (up- or down-regulated).** FabI-specific probes are highlighted in yellow. In bold, the mean value of the fold increase/decrease. Gene are presented in the decreasing fold change for the upregulated gene and in increase fold change for the downregulated genes. The fold change values of every single probe are also indicated. Probes are described as follow: Probe ID/Probe sequence/Gene Name/NCBI Protein Reference Sequence number/Protein product name. Additional file 3:
**Supplementary data S3: detailed informations on the genes commonly up- or down-regulated in the 4 clinical** ***S. aureus*** **characterized by a reduced susceptibility to triclosan.** Described are the gene locus and Uniprot ID as found for the TW20 or MU50 genomes, as well as the associated KEGG orthology and gene ontologies (GO) when available. GO terms were organized in 3 categories: “Biological Process”, “Molecular Function” and “Cellular Component”. Additional file 4:
**Supplementary data S4: list of genome sequences and respective accession numbers used for probes design.**

## Abbreviations

ACP: Enoyl-acyl carrier protein
NAD: Nicotinamide adenine dinucleotide
RND: Resistance-nodulation-division
SNP: Single nucleotide polymorphism
IS256: Insertion sequence element 256
MIC: Minimal inhibitory concentration
MBC: Minimal bactericidal concentration
GO: Gene ontology
CCCP: Carbonyl cyanide-m-chlorophenylhydrazone
MRSA: Methicillin-resistant *Staphylococcus aureus*
TSB: Tryptic soy broth
INDELs: Insertions and deletions
